# Quality of Life of Developmentally Normal Children With Epilepsy and Their Siblings

**DOI:** 10.7759/cureus.44067

**Published:** 2023-08-24

**Authors:** Abhishek Navik, Anju Aggarwal, Aaradhana Singh, Rajeev Kumar Malhotra

**Affiliations:** 1 Pediatrics, University College of Medical Sciences and Guru Tegh Bahadur Hospital, New Delhi, IND; 2 Statistics, All India Institute of Medical Sciences, New Delhi, IND

**Keywords:** peds ql, qolce 55, quality of life, siblings, childhood epilepsy

## Abstract

Background: Quality of life (QOL) is a fundamental and multidimensional concept that should be considered with health problems, specifically chronic diseases, such as epilepsy. There have been limited studies on how pediatric epilepsy impacts the QOL of siblings of affected individuals. Hence, we studied the impact of epilepsy on the QOL of affected children and their siblings.

Objective: This study aimed to assess the QOL of developmentally normal children with epilepsy and their siblings and the association of QOL scores with the clinicodemographic profile.

Methods: This study was conducted at the University College of Medical Sciences and Guru Tegh Bahadur Hospital, New Delhi, India, a tertiary care hospital. The QOL of children (4-12 years) with epilepsy was assessed using epilepsy-specific questionnaires, i.e., Quality of Life in Childhood Epilepsy Questionnaire-55 (QOLCE-55), which covers the cognitive, emotional, social, and physical domains, and Pediatric Quality of Life Epilepsy Module (Peds QL EM), which covers the impact, cognitive, sleep, executive, and mood/ behavior domains. QOL in siblings was assessed using the Peds QL Inventory, which covers the following domains: physical, emotional, social, and school. The principal investigator administered these questionnaires to parents in Hindi/ English. Scoring was done as per standard instructions of the questionnaire. Clinical and demographic data were recorded in a pro forma.

Result: The median QOLCE-55 score was 81.12, with a range of 74.65-86.34, and the median Peds QL EM score was 89.31, with a range of 75.58-94.48. Overall, Cronbach's alpha of QOLCE-55 and Peds QL EM was >0.8. Breakthrough seizures (≥10) affected the overall QOL (p=0.001) and all domains of QOLCE-55 (except emotional function (p=0.44)) and Peds QL EM (except sleep/fatigue domain (p=0.59)). Age, sex, parental education, socioeconomic status, and type of epilepsy did not affect the overall QOL (p>0.05). The QOL of siblings was not affected as per the Peds QL Inventory score (median score 100) and self-made questionnaire.

Conclusion: Our results suggest that the QOL of children with epilepsy was compromised, whereas the QOL of their siblings was not affected.

## Introduction

Quality of life (QOL) is a fundamental and multidimensional concept that should be considered in health problems [1]. It is a crucial tool for assessing the impact of disease and treatment interventions; as a result, it can be employed as the primary outcome indicator and a determinant of treatment benefit [2,3]. Epilepsy is a chronic disorder in which seizures may take several years to control. Several epileptic and nonepileptic factors can affect the QOL in children with epilepsy. Moreover, the diagnosis of a chronic illness, such as epilepsy, during childhood is a source of severe stress and poor QOL for the whole family, including parents and siblings [4]. Although few Indian studies [5-11] have tried to ascertain the QOL of children with epilepsy, there are limited studies on how pediatric epilepsy impacts the QOL of siblings [12-15]. Hence, we have attempted to assess the QOL of children with epilepsy using epilepsy-specific questionnaires Quality of Life in Childhood Epilepsy Questionnaire-55 (QOLCE-55) and Pediatric Quality of Life Epilepsy Module (Peds QL EM), and the QOL of their siblings was evaluated using the Peds QL Inventory.

## Materials and methods

The study was conducted in University College of Medical Sciences and Guru Tegh Bahadur Hospital, a tertiary care hospital in New Delhi, India, between January 2021 and April 2022. A sample size of 80 was calculated using a standard formula using the mean and standard deviation (62.62 ± 21.32) of the QOL score from a study by Aggarwal et al. [5], which used QOLCE questionnaires. Eighty developmentally normal children with generalized or focal motor epilepsy under treatment from the author's institution in the age group of 4 to 12 years and their closest siblings (4 to 18 years) living in the same house were enrolled in the study. For the study, epilepsy was defined as "having at least two unprovoked (or reflex) seizures occurring greater than 24 hours apart or one unprovoked (or reflex) seizure and a probability of further seizures similar to the general recurrence risk (at least 60%) after two unprovoked seizures, occurring over the next 10 years" [16]. "Developmentally normal" was taken as the achievement of age-appropriate milestones and/or studying in an age-appropriate class as per history and examination. Subjects with comorbid severe chronic disease, genetic syndromes, myoclonic seizures, and absence seizures and families with multiple children with epilepsy were excluded from the study. Ethical approval was taken from the Institutional Ethics Committee of the University College of Medical Sciences, New Delhi, India (approval number: IECHR/2020/PG/46/61), and informed written consent/assent was taken from the participants and their parents.

Data collection

Demographic and clinical details were noted in the case record form. Parents of the participating children were informed and explained the importance of assessing the QOL of the children, and we proposed to evaluate it using standard questionnaires (QOLCE-55, Peds QL EM, and Peds QL Inventory). It was administered to them in a language (Hindi/English) they understood and were guided by the chief investigator to fill it. The standard instructions were followed while filling out and calculating the questionnaire scores.

We also administered a self-formulated parent-answered questionnaire that reflected on the day-to-day activities of the siblings, their nutrition, and schooling, and their responses were also noted in the case record form.

Instruments

The questionnaires used in the study were translated into Hindi by medical and non-medical experts. Furthermore, the translated version of the questionnaire was retranslated into English to ensure exact meaning of the original version. We used the English/Hindi translated version of QOLCE-55 [17,18], for assessing the QOL of children with epilepsy. The questionnaire has four domains: cognitive (22 items), emotional (17 items), social (seven items), and physical (nine items). We also used the English/Hindi-translated parent proxy versions of Peds QL EM [19,20], which has five domains: impact (nine items), cognitive (six items), sleep (three items), executive (six items), and mood/behavior (five items).

The QOL of siblings was assessed using the English/Hindi translated version of the parent proxy Peds QL Inventory for the age group 4-18 years. The Peds QL Inventory [21] contains 23 items that make up the following scales: physical (eight items), emotional (five items), social (five items), and school (five items).

Statistical analysis

Data analysis was done with IBM SPSS Statistics for Windows, version 20 (released 2011; IBM Corp., Armonk, New York, United States). An overall QOL score was computed by adding each subscale score for each individual and then dividing it by the total number of items answered. Individual domain scores were calculated by adding the scores of questions answered and dividing them by the respective number of questions answered. Higher scores were indicative of a better QOL. Cronbach's alpha was used to determine the internal consistency and reliability of the Peds QL EM and QOLCE-55 subscales. A value >0.700 is considered indicative of good internal consistency reliability. The Shapiro-Wilk test was used to determine whether the QOL scores were normally distributed. As the QOLCE-55 and Peds QL EM scores violated the normality condition, we used non-parametric methods to assess the relationship between QOL scores and seizure characteristics and demographic variables. Mann-Whitney U test was used to compare the distribution of QOL scores between the sex of the patient, socioeconomic status of patients, type of seizures, and frequency of seizures, and the Kruskal-Wallis test was used to compare the distribution of QOL scores among maternal education. Spearman's correlation was applied to find the strength of the correlation between the QOL scores and age and duration of seizures of patients. P value <0.05 was considered statistically significant.

## Results

A total of 80 developmentally normal children with epilepsy (4-12 years) and their siblings (4-18 years ) were evaluated using QOLCE-55, Peds QL EM, and Peds QL Inventory. Table 1 shows their demographic data and clinical characteristics.

**Table 1:** Clinical characteristics of the study subjects

Clinical characteristics	N(%)
Age of patients (years)	
4-6	21(26.2)
7-9	32(40)
10-12	27(33.7)
Age of siblings (years)	
4-6	16(20)
7-9	20(25)
10-12	25(31.2)
13-18	19(23.7)
Sex of patients	
Male	50(62.5)
Female	30(37.5)
Sex of siblings	
Male	38(47.5)
Female	42(52.5)
Seizure type	
Generalized	58(72.5)
Focal	22(27.5)
Number of anti-seizure drugs	
Single	73(91.2)
Multiple	7(8.7)
Name of anti-seizure drug	
Valproate	55(68.7)
Phenytoin	11(13.7)
Levetiracetam	1(1.25)
Carbamazepine	6(7.5)
Polytherapy	7(8.7)

Of the 80 children with epilepsy, 62 (77%) underwent CT or MRI. Most of them had normal imaging reports. Neurocysticercosis was seen in seven and tuberculoma in three. Since the study was conducted during COVID times, cross-sectional EEG was available only in 10 patients, out of which six were abnormal. The mean age of the patients was 8.23±2.47 years. The mean age of the siblings was 10.15±3.87 years. Of the 80 siblings, 23 were younger siblings, and 57 were older siblings. Most parents were educated until high school (46%) and belonged to the upper-lower class (88.7%) as per the modified Kuppuswamy Scale 2021 [22]. Most of the patients in our study had generalized seizures (72.5%) with fewer than 10 breakthrough seizures. Most were on a single anti-seizure drug (73), and seven were on multiple anti-seizure medications (Table 1).

The median QOLCE-55 score was 81.12, with an interquartile range (74.65-86.34) (Table 2). Physical functioning was affected with the lowest median of 46.42 (39.28-59.82). The median Peds QL Epilepsy score was 89.31 (75.58-94.48). The impact score was affected with the lowest median of 80.20 (71.87-87.50). A value of >0.7 for Cronbach's alpha indicates good internal consistency reliability. Overall, Cronbach's alpha for QOLCE-55 and Peds QL EM was >0.8, with the highest being in the cognitive function domain (>0.8).

**Table 2:** Median, interquartile ranges, and Cronbach's alpha of QOLCE-55 and Peds QL EM scores. Spearman's correlation of age and sex of patients with QOL scores. *Spearman correlation. **P value is significant at the 0.05 level. IQR: interquartile range, QOL: quality of life, QOLCE-55: Quality of Life in Childhood Epilepsy Questionnaire-55, Peds QL EM: Pediatric Quality of Life Epilepsy Module

Questionnaires	Median and IQR of QOLCE-55 and PED QL EM	Cronbach's alpha of QOLCE-55 and PEDS QL EM	Relation of age of patient with QOL	Relation of duration of seizures with QOL
QOLCE-55 cognitive function	97.67 (88.66-100)	0.855	-0.12 (P=0.27)*	-0.12 (P=0.28)*
QOLCE-55 emotional function	93.75 (87.50-100)	0.632	-0.037 (P=0.74)*	0.12 (P=0.29)*
QOLCE-55 social function	92.86 (78.57-100)	0.675	-0.056 (P=0.62)*	0.07 (P=0.52)*
QOLCE-55 physical function	46.42 (39.28-59.82)	0.566	0.228 (P=0.042)**	-0.056 (P=0.59)*
QOLCE-55 total score	81.12 (74.65-86.34)	0.831	0.02 (P=0.83)*	0.10 (P=0.35)*
Peds QL EM impact	80.20 (71.87-87.50)	0.607	-0.07 (P=0.53)*	-0.06 (P=0.58)*
Peds QL EM cognitive function	89.58 (60.62-100)	0.828	-0.12 (P=0.28)*	-0.11 (P=0.31)*
Peds QL fatigue/sleep	100 (75-100)	0.485	-0.50 (P=0.66)*	0.03 (P=0.79)*
Peds QL executive function	97.91 (50-100)	0.794	-0.11 (P=0.31)*	0.13 (P=0.24)*
Peds QL mood/behavior	90.00 (60-100)	0.733	0.02 (P=0.83)*	-0.05 (P=0.64)*
Peds QL total score	89.31 (75.58-94.48)	0.882	-0.06 (P=0.54)*	-0.13 (P=0.24)*

**Figure 1:**
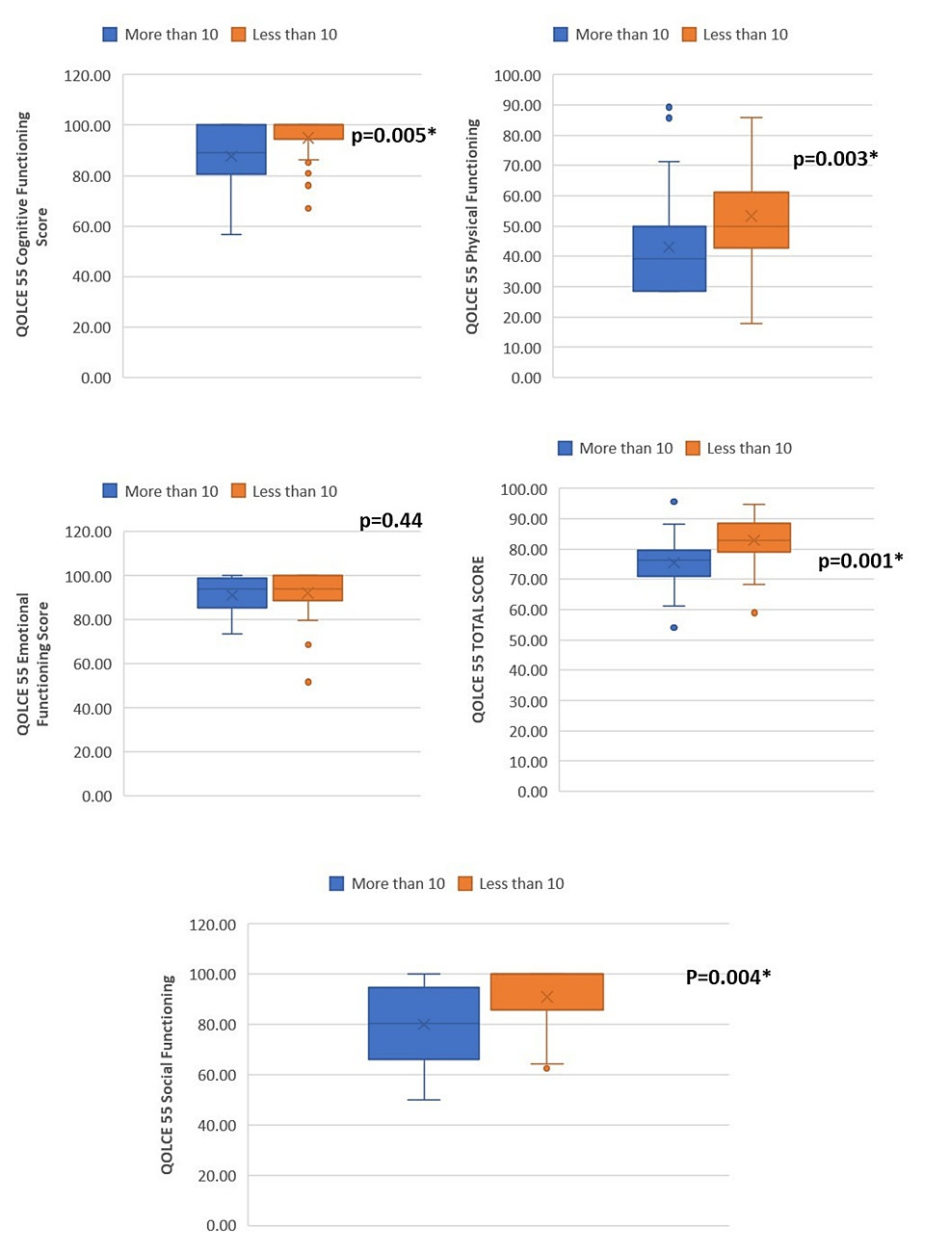
Relation of number of breakthrough seizures with Quality of Life in Childhood Epilepsy Questionnaire-55 (QOLCE-55) scores

**Figure 2:**
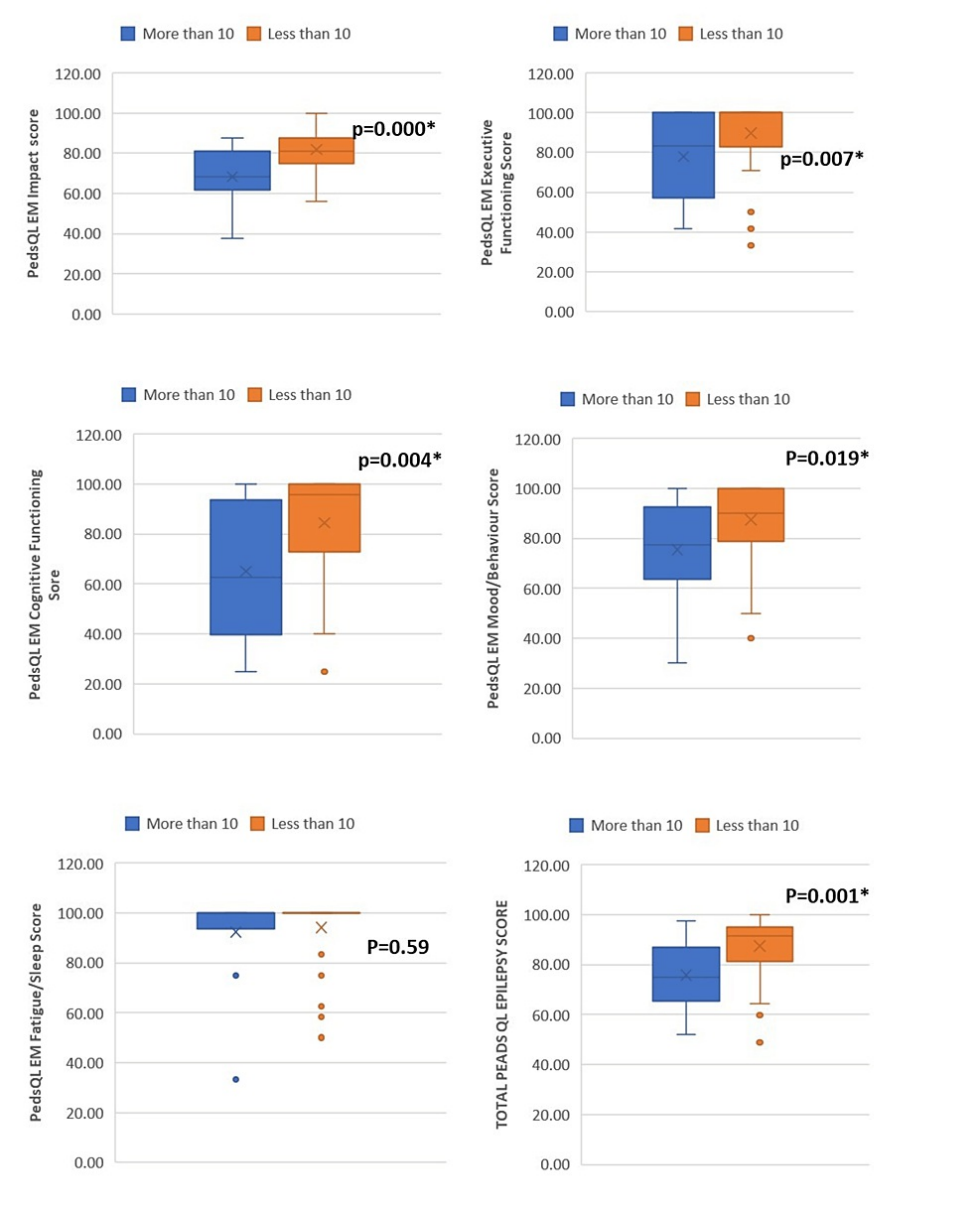
Relation of Pediatric Quality of Life Epilepsy Module (Peds QL EM) scores with the number of breakthrough seizures

The number of breakthrough seizures (≥10) affected the total (p=0.001) and individual domains of QOLCE-55 and Peds QL EM (except QOLCE emotional function (p=0.44) and Peds QL EM sleep/fatigue domain (p=0.59)) (Tables 3, 4 and Figures 1, 2).

**Table 3:** Relation of the QOLCE-55 scores (median with the interquartile range) with various clinical characteristics of the patient * Man-Whitney Test; # Kruskal-Wallis Test; ** P-value is significant at the 0.05 level. QOLCE-55: Quality of Life in Childhood Epilepsy Questionnaire-55

QOLCE-55	Sex		P*	Maternal education				P^#^	Socioeconomic status		P*	Type of seizures		P*	Frequency of seizures		P*
	Male N=50	Female N=30		Illiterate N=13	1st-5thN=11	5th-12th N=46	Graduate N=10		Upper- and lower-middle class N=9	Upper-lower class N=71		Generalized N=58	Focal N=22		≥10 N=22	<10 N=58	
Cognitive function	97.67 (86.34-100)	98.81 (92.98-100)	0.50	100 (87.44-100)	97.73 (86.36-100	97.49 (91.07-100)	98.30 (89.45-100)	0.91	100 (79.55-100)	97.62 (88.75-100)	0.94	96.98 (87.19-100)	100 (90.94-100)	0.26	89.02 (80.45-100)	100 (94.35-100)	0.005**
Emotional function	93.75 (85.93-100)	93.75 (88.67-98.82)	0.98	92.17 (85.46-96.92)	93.75 (87.50-96.87)	95.31 (87.50-100)	93.75 (83.83-100)	0.86	85.93 (82.23-93.75)	95.31 (87.50-100)	0.03**	94.53 (85-98.89)	93.75 (89.76-100)	0.39	94.53 (85-98.89)	93.75 (88.67-100)	0.44
Social function	91.07 (77.68-100)	98.21 (78.57-100)	0.79	85.71 (80.36-97.92)	92.86 (87.50-100)	96.43 (71.43-100)	100 (83.04-100)	0.54	85.71 (78.57-100)	92.86 (78.57-100)	0.66	89.29 (78.57-100)	100 (78.57-100)	0.31	80.36 (66.07-94.64)	100 (85.71-100)	0.004**
Physical function	46.42 (39.82-58.48)	50.00 (37.50-60.71)	0.95	46.42 (42.87-64.28)	53.57 (33.33-71.42)	44.64 (33.33-57.14)	47.50 (42.85-71.42)	0.63	42.85 (30.03-60.71)	46.42 (39.28-60.71)	0.33	46.42 (39.82-57.14)	48.21 (33.33-63.39)	0.86	39.28 (28.57-50)	50 (42.85-61.16)	0.003**
Total score	81.22 (75.81-86.27)	81.62 (73.58-88.19	0.89	81.47 (75.11-86.92)	81.19 (76.27-91.18)	80.00 (74.22-86.18)	85.20 (70.08-90.0)	0.80	79.12 (69.22-85.31)	81.25 (76.19-87.50)	0.20	80.64 (73.88-86.12)	83.00 (75.74-88.32)	0.27	76.23 (71.10-79.53)	83.00 (78.93-86.64)	0.001**

**Table 4:** Relation of PEDS QL EM scores (median with interquartile range) with various clinical characteristics of the patient. * Man-Whitney test; # Kruskal-Wallis test; ** P-value is significant at the 0.05 level. PEDS QL EM: Pediatric Quality of Life Epilepsy Module

PEDS QL EM	Sex		P*	Maternal education				P^#^	Socioeconomic status		P*	Type of seizures		P*	Frequency of seizures		P*
	Male N=50	Female N=30		Illiterate N=13	1st-5th N=11	5th-12th N=46	Graduate N=10		Upper- and lower-middle class N=9	Upper-lower class N=71		Generalized N=58	Focal N=22		>10 N=22	<10 N=58	
Impact	81.25 (71.42-87.50)	78.64 (71.87-87.50)	0.88	78.12 (71.87-85.93)	81.25 (68.75-87.50)	76.64 (68.75-87.50)	87.50 (74.21-87.50)	0.45	81.25 (70.31-87.50)	79.16 (71.87-87.50)	0.89	78.12 (71.42-87.50)	81.25 (74.21-87.50)	0.32	68.30 (61.71-81.25)	81.25 (75-87.50)	0.00
Cognitive function	85.41 (61.87-100)	91.66 (58.33-100)	0.52	83.33 (57.08-100)	66.66 (54.16-100)	91.66 (62.50-100)	100 (65.00-100)	0.31	100 (61.12-100)	87.50 (60-100)	0.21	87.50 (60-100)	91.66 (64.58-100)	0.76	62.50 (39.58-93.75)	95.83 (72.91-100)	0.004**
Fatigue/sleep	100 (100-100)	100 (100-100)	0.97	100 (100-100)	100 (100-100)	100 (83.33-100)	100 (100-100)	0.24	100 (100-100)	100 (100-100)	0.58	100 (95.83-100)	100 (100-100)	0.05	100 (93.75-100)	100 (100-100)	0.59
Executive function	91.66 (70.83-100)	100 (83.33-100)	0.07	87.50 (77.08-100)	87.50 (79.16-100)	97.91 (73.95-100)	100 (91.66-100)	0.55	100 (75-100)	95.83 (79.16-100)	0.74	97.91 (78.15-100)	95.83 (79.16-100)	0.87	83.33 (57.29-100)	100 (82.81-100)	0.007**
Mood/behavior	90.00 (70-100)	90.00 (73.75-100)	0.98	90 (62.50-100)	90 (75-100)	90 (70-100)	100 (90-100)	0.41	90.00 (87.50-100)	90.00 (70-100)	0.26	90.00 (70-100)	90.00 (83.75-100)	0.33	77.50 (63.75-92.50)	90.00 (78.75-100)	0.019**
Total score	90.01 (71.13-94.62)	87.02 (80.26-95.12)	0.55	89.93 (69.10-93.06)	86.33 (74.62-94.28)	87.16 (75.03.22-95.00)	93.75 (88.38-97.50)	0.25	93 (77.72-96.50)	88.88 (75.57-94.28)	0.34	87.58 (73.19-94.62)	91.16 (80.16-95.12)	0.43	75.09 (65.58-86.68)	91.58 (81.14-95.12)	0.001**

Demographic characteristics, such as age, sex, socioeconomic status, and parental education, and seizure characteristics, such as type epilepsy and duration of epilepsy, did not affect the individual domain and overall QOLCE-55 and Peds QL EM scores. However, the QOLCE physical functioning scores were significantly lower in the younger age groups (p=0.04), and the QOLCE emotional function scores were significantly better in the upper-lower class compared to the upper- and lower-middle class (p=0.03) (Tables 2, 3, 4).

The siblings' median Peds QL Inventory score in our study was 100. We also administered self-formulated parent-answered questionnaires to study the impact of childhood epilepsy on the siblings. Their responses are shown in Table 5.

**Table 5:** Answers to the self-formulated parent-answered questionnaires to study the impact of childhood epilepsy on the siblings NA: Not Applicable

Self-formulated parent-answered questionnaires	Response		
	Yes	No	N/A
1. Does your child feel that their well-being is affected due to their sibling's condition?	2	78	0
2. Does your child think that they have to do more household work because of their sibling?	12	67	1
3. Does your child play with their sibling?	80	0	0
4. Do your child's friends bully/avoid/tease them because of their sibling?	0	77	3
5. Is your child comfortable taking their sibling to friends' gatherings?	62	6	12
6. Do you think you have certain social commitment restrictions because of your sibling?	3	67	10
7. Do you think other kids don't want to be your child's friend because of their sibling?	1	75	4
8. Do you feel your child gets scolded more than usual because of a sibling?	11	67	2
9. Is your child afraid/scared of their sibling?	7	73	0
10. Does your child get sufficient food for all meals?	80	0	0
11. Does your child like the variety of food prepared?	78	2	0
12. Does your child require your help with feeding?	3	77	0
13. Does your child feel that the food of his choice is not prepared in sufficient frequency?	25	52	3
14. Do teachers complain about your child's school performance?	5	67	8
15. Do you feel the time allotted to your child by you (parents) for their homework is sufficient?	60	2	18
16. Does your child attend the tuition class?	34	43	3
17. Does your child miss school because of their sibling's health condition?	8	64	8
18. Does your child miss school because of any chronic/recurrent disease?	0	65	15
19. Does your child have a problem paying attention in class?	4	65	11
20. Does your child complete their homework regularly?	69	2	9
21. Does your child feel angry that you (parents) pay more attention to their sibling?	6	71	3

## Discussion

The QOL among chronic disease patients is compromised significantly, especially in children with epilepsy. In this study, we measured QOL using two scales, QOLCE-55 and Peds QL EM, in 80 developmentally normal children with generalized or focal motor epilepsy in the age group of 4-12 years. In siblings of children with epilepsy, the QOL may be compromised due to domestic, social, and cultural issues. Their QOL was assessed using the Peds QL Inventory scale. In our study, the overall QOLCE-55 scores (median of 81.12 with an interquartile range of 74.65-86.34) were reasonable compared to other Indian studies, which used the same questionnaire (Pachange et al., 69.9±13.43; Nagesh et al., 48.20±10.68) [7,8]. This could be because our patients were from an urban area and receiving treatment from a tertiary center with cost-free anti-seizure drugs and epilepsy-related investigations. Moreover, the majority of our patients had well-controlled epilepsy. The overall Peds QL EM score was 89.31 (75.58-94.48); a direct comparison of this score was not possible as no other Indian studies used this scale. In our study, we found that the number of breakthrough seizures (≥10) is the factor that affected not only the overall QOL (p=0.001) but also almost all the domains of QOLCE-55 (except emotional function (p=0.44)) and Peds QL EM (except sleep/fatigue domain (p=0.59)). The highly unpredictable nature of the disease could easily explain this. Seizure recurrence is unpredictable, posing a constant threat to epilepsy patients and their families.

A similar finding was seen in other Indian studies by Aggrawal et al. [5], Pachange et al. [7], Nagesh et al. [8], and Nadkarni et al. [10]. Meanwhile, Lagunju et al. [9] and Arya et al. [23] observed no effect of frequency of seizures on QOL scores. We also observed that age, sex, parental education, family socioeconomic status, and type of epilepsy did not affect the overall QOL scores (p>0.05). Our findings match those of Arya et al. [23], who found that parental education, socioeconomic status, or type of epilepsy did not affect the overall QOL of children with epilepsy. Even Pachange et al. [7] demonstrated no relation between the overall QOL with parental education, type of epilepsy, and socioeconomic status, but they found that the patient's age significantly affected the QOL. Srujna et al. [11] observed that gender and age of participants had no relation to the QOL, but factors, such as maternal education and socioeconomic status, strongly correlated with QOL. Haider et al. [6] observed that the type of epilepsy had no relation to QOL, but the patient's age and gender affected QOL. In contrast to other studies, such as Nadkarni et al. [10], they observed that the QOL was more affected in older children with lower socioeconomic status and mothers with lower literacy levels. Even Aggrawal et al. [5] observed that the QOL was affected by age, maternal education, and type of epilepsy. Moreover, a study by Langunju et al. [9] demonstrated the relationship of the overall QOL with maternal education. This disparity among studies could be attributable to the type of questionnaire (generic/disease-specific), differing age groups of children recruited, increased awareness of epilepsy through time, and improved treatment availability. The physical functioning domain (p=0.003) of QOLCE-55 and the impact domain (p=0.00) of Peds QL EM were the most affected domains in our study. The questions in both domains assess how epilepsy interferes with daily activities, peer interaction, independence, and increased disease burden due to treatment. Pachange et al. [7] found a similar result when they utilized the QOCLE-55 scale to evaluate the QOL of 108 children with epilepsy (ages 13-15), with physical functioning being the most impaired domain. However, Aggarwal et al. and Nagesh et al. [5,8] discovered that cognitive function was the most impaired domain. The behavior, social, and emotional domains were the most influenced according to Nadkarni et al. [10], whereas self-esteem was the most affected according to Arya et al. [23].

Although our finding of the physical functioning domain being most affected differed from those of most other studies, our results could be explained by the fact that most children in our study were between the ages of four and nine and that a large majority of the caregivers were illiterate, causing them to perceive higher risks for their children and misinterpret information about their children's conditions. Essentially, they did not fully grasp their children's state or were not informed well enough. Therefore, they can be more restricted in their children's different activities.

Our study also found better QOLCE-55 emotional function scores in the upper-lower class compared to the upper- and lower-middle-class patients (p=0.03). The emotional function was impaired in the upper- and lower-middle classes, most likely due to over-stigmatization of the disease and too much concerned parents, which reflected on the children's QOL.

Our study's overall QOL of siblings with epilepsy was good, with a Peds QL Inventory median score of 100. Moreover, the self-formulated parent-answered question responses reflected no effect of childhood epilepsy on most siblings regarding their schooling, nutrition, activity, and day-to-day affairs (Table 5). No similar studies used this scale, so a direct comparison was not possible. Several international studies have attempted to interview families of children with epilepsy to measure the impact of epilepsy on siblings and have found that epilepsy has a significant influence on the siblings' QOL [12,13,24].

The QOL of the siblings was unaffected in our study because most of our patients had well-controlled epilepsy and were developmentally normal. Siblings have a unique affinity in the Indian society because they spend more time together as youngsters than they do with their parents or anybody else. Most Indian families have deep relationships between siblings and family members, offering stability and values, such as sharing, caring, empathy, and understanding.

Limitations

Our study had a limitation of children having predominantly well-controlled epilepsy. Hence, further studies need to be carried out on larger sample sizes and equitable distribution of well, moderate, and poorly controlled epilepsy in different populations to assess the QOL of siblings in children with epilepsy. The questionnaires used in our study were standard, although Peds QL EM questionnaires were validated in Indian children for the first time. The questionnaires used in the study were parent-reported. At times, reports made by parents may not reflect the views of the children.

## Conclusions

Epilepsy-specific questionnaires Peds QL EM and QOLCE-55 are valid for assessing the QOL in Indian children with epilepsy. Children with epilepsy have compromised QOL, the most affected domain being the physical domain. The QOL of siblings of these developmentally normal children with epilepsy was not much affected. These findings have implications for physicians caring for children with epilepsy and their siblings.
